# Positive STEPS – a randomized controlled efficacy trial of an adaptive intervention for strengthening adherence to antiretroviral HIV treatment among youth: study protocol

**DOI:** 10.1186/s12889-018-5815-9

**Published:** 2018-07-13

**Authors:** Matthew J. Mimiaga, Lisa M. Kuhns, Katie B. Biello, Jennifer Olson, Sam Hoehnle, Christopher M. Santostefano, Jaclyn M. W. Hughto, Hadeis Safi, Peter Salhaney, Diane Chen, Robert Garofalo

**Affiliations:** 1 Center for Health Equity Research, Brown University, 121 South Main Street, Providence, RI 02903 USA; 2 Departments of Behavioral & Social Health Sciences and Epidemiology, Brown University School of Public Health, Providence, RI USA; 3 Department of Psychiatry and Human Behavior, Brown University Alpert Medical School, Providence, RI USA; 4 The Fenway Institute, Fenway Health, Boston, MA USA; 5 Division of Adolescent Medicine, Ann & Robert H. Lurie Children’s Hospital of Chicago, Chicago, IL USA; 6 Feinberg School of Medicine, Department of Pediatrics, Northwestern University, Chicago, IL USA

**Keywords:** Adherence, Antiretroviral therapy (ART), HIV treatment, Youth

## Abstract

**Background:**

HIV infection among youth in the United States is on the rise. A high level of antiretroviral therapy (ART) adherence is crucial to treatment success and can minimize the population burden of the disease. However, the overall rate of ART adherence among youth is generally suboptimal and no published efficacious interventions exist to address the specific needs of this population. This paper describes the design of a stepped-care, “adaptive” ART adherence intervention protocol for HIV-infected adolescents and young adults.

**Methods:**

This is a randomized controlled trial (RCT) to establish the efficacy of “Positive STEPS,” a behavioral and technology-based intervention to optimize ART adherence and viral suppression among HIV-infected youth, ages 16 to 29. Participants are equally randomized to 1) the Positive STEPS intervention, which begins with two-way daily text messaging as a reminder system to take their medications; participants progress to a more intensive in-person counseling intervention if text messaging is not sufficient to overcome barriers; or 2) or standard of care (SOC). At randomization, all participants receive standardized ART adherence education. During the 4 major study assessment visits (baseline, 4-, 8-, and 12-months), participants have their blood drawn to measure HIV viral load and complete a mix of computer-based self-administered and interviewer-administered behavioral and psychosocial measures. The primary outcomes are improvements in viral load and ART adherence measured via a medication-tracking device (i.e., Wisepill) and self-report.

**Discussion:**

Behavioral interventions are greatly needed to improve ART adherence among HIV-infected adolescents and young adults and prevent onward transmission. If effective, the intervention tested here will be one of the first rigorously-designed efficacy trials to promote ART adherence in this population, using an approach that holds promise for being readily integrated into real-world clinical settings.

**Trial registration:**

ClinicalTrials.gov number NCT03092531, registered March 28, 2017.

## Background

A significant and growing population of youth are acquiring and living with HIV in the United States. According to the CDC, more than16,000 youth (aged 13 to 29) were diagnosed with HIV in 2015, accounting for over 40% of all new HIV infections in the U.S. [1, 2]. Approximately 80% of these diagnoses occurred in persons aged 20 to 29 years, who, strikingly, had the highest rate of HIV infections of any age group (65 new HIV diagnoses/100,000 people) [2]. Advances in medical treatment, specifically antiretroviral therapy (ART), has resulted in precipitous declines in HIV-associated morbidity and mortality [3–6], allowing for HIV-infected adolescents and young adults to manage their HIV as a chronic disease. However, maintaining high levels of adherence (85–95%) is crucial to treatment success [7, 8], and promoting adherence remains an essential element in modern HIV care [9, 10].

While ART confers many health benefits, consistent long-term ART adherence can often be challenging for HIV-infected adolescents, and the overall rate of ART adherence in this age group is generally suboptimal. Across 22 published studies [11], ART adherence among adolescents ranged from 28 to 69%, much lower than the 85–95% required to optimize treatment gain [12]. In addition, a large-scale progression study in the U.S. of HIV-infected adolescents found that only 41% of adolescents on ART reported > 95% adherence [13] despite substantial advances made to simplify regimens. Behaviors associated with adherence (e.g., taking doses at the same time every day, not skipping doses due to irregularity in routines) remain challenging, especially for young people with HIV [14]. The typical trajectory of adolescence and young adulthood involves behavioral experimenting, risk taking, and confronting a host of difficult choices with regard to romantic relationships, sexual behavior, substance use, and identity formation [15]. The complexity of these factors is compounded for HIV-infected adolescents and emerging adults (18 to 29 year olds) who must negotiate their lives within the framework of having a chronic and stigmatizing disease [16–18]. Further, developmental cognitive processes, such as concrete thinking and partially developed abstract reasoning, may contribute to difficulties in taking medications for adolescents who are asymptomatic, particularly if the medications have adverse side effects and serve to differentiate them from peers [19, 20].

Given these considerations, the most promising strategies for improving treatment adherence among HIV-infected adolescents and young adults may involve multiple components such as patient education, self-monitoring, and medication-taking reminders. The need for multi-level components is consistent with adult adherence interventions, where multiple strategies tend to be most effective in improving adherence [21, 22]. There is a paucity of interventions aimed at improving ART adherence among HIV-infected adolescents and young adults and only one that meets Center for Disease Control and Prevention (CDC) minimal evidence for strength of evidence [23]. Among the few pilot trials of interventions that have been conducted, two required significant study resources and staff time to conduct home visits and monitor directly-observed therapy, raising concerns about sustainability in real world settings [24, 25]. Other pilot studies of ART adherence interventions among HIV-infected adolescents and young adults addressed only one barrier to adherence, such as pill swallowing [26] or a reminder system to take their medications [27]. Furthermore, while six prior intervention pilot trials to enhance ART adherence among HIV-infected adolescents and young adults have been conducted that incorporate education and skills building and resulted in modest effects [28–33], no efficacy data from a full-scale trial have been published to date. Only one intervention has been tested which meets CDC evidence of efficacy: a text-message based reminder intervention [34] on which we are building for our stepped care approach. As such, developing interventions that are acceptable, feasible to deliver, and responsive to the unique ART adherence challenges and contextual realities experienced by HIV-infected adolescents and young adults are needed.

For the current study, a stepped-care model [35] is used for improving ART adherence among HIV-infected adolescents and young adults. Under this model, the least resource intensive intervention is delivered first, and only those who do not improve receive the high-intensity, more resource intensive intervention. This approach has the explicit intent of applying resources efficiently, while still providing patients with effective interventions. The Positive STEPS intervention starts with and capitalizes on the increasingly widespread use of technology among adolescents and young adults by including text message reminders as social cues to facilitate ART adherence, building on prior evidence of efficacy of this approach [34]. Participants progress to a more intensive in-person counseling intervention if text messaging is not sufficient to overcome barriers. Modeled after Life-Steps [36], an efficacious ART adherence intervention for HIV-infected adults, the current intervention is based on principles of cognitive-behavioral therapy [37], motivational interviewing [38], and problem solving [39, 40] and addresses 11 steps over five, in-person counseling sessions with a master’s-level counselor.

The present paper describes the Positive STEPS intervention in more detail, and provides an overview of the methods for efficacy testing. The specific aims of this study are to (1) determine the efficacy of the Positive STEPS intervention in comparison to a standard-of-care (SOC) condition on viral load suppression and improvement of ART adherence among HIV-infected adolescents, ages 16–29; and (2) examine the degree to which viral load suppression and improvement ART adherence occur in the context of theoretical mediators of our conceptual model of intervention effects (e.g., social-support, self-efficacy, motivation, self-regulation) and potential moderators (e.g., depression, anxiety, substance use, stigma) of the intervention.

## Methods/design

### Overview of design

**Fig. 1:**
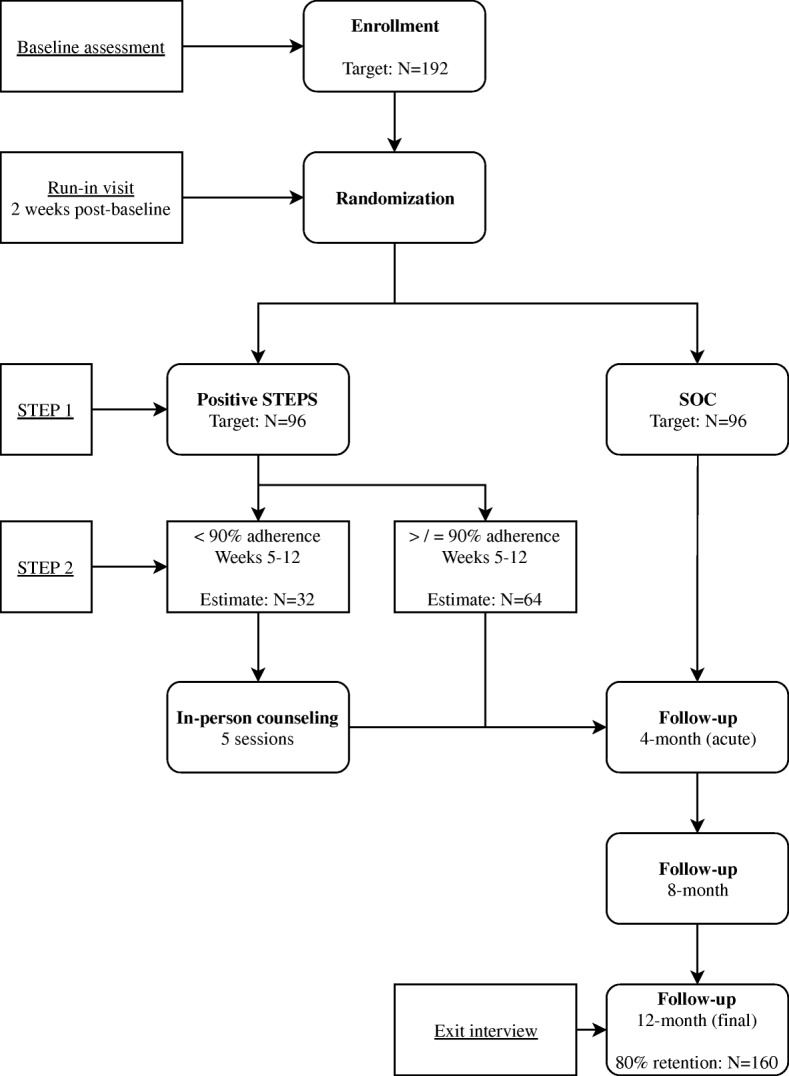
Flow chart of study process

This a two-arm randomized controlled trial to examine the efficacy of a stepped-care intervention, Positive STEPS, which includes daily two-way text messaging (Step 1) and a five-session face-to-face manualized intervention (Step 2) compared to a SOC condition. Participants in both arms monitor their ART adherence using Wisepill technology, which makes use of mobile phones and internet technologies to provide real-time medication monitoring on the Wisepill website using a standard internet browser. During the baseline visit, participants are given and shown how to use the Wisepill throughout the course of the study. The duration of participation is 12 months with four major assessment points: baseline, 4- (acute outcome), 8-, and 12-months (Fig. 1).

### Identification and recruitment of participants

The current study aims to enroll 192 HIV-infected adolescents and young adults, ages 16–29, over three years at study sites in Providence, Rhode Island (*N* = 96) and Chicago, Illinois (N = 96). Participants are recruited via outreach to local medical centers, colleges and universities, community-based organizations (e.g., AIDS service organization), events (e.g., PRIDE), and social media outreach and advertisement (e.g., Facebook, Craigslist). Inclusion and exclusion criteria for participation are shown in Table 1.

**Table 1:** Inclusion and exclusion criteria

Inclusion Criteria	Exclusion Criteria
1) 16–29 years old (inclusive)	Enrolled in another ART adherence intervention study
2) HIV-infected (verified at baseline)	
3) Taking/prescribed ART ≥3 months	Unable to give informed consent due to mental/physical illness, cognitive limitation, or substance intoxication
4) Self-reported difficulties (< 90%) adhering to ART in past month (i.e., missed ≥4 doses in past month OR ≥ 1 dose in past week)	
5) Owns/has daily access to a cell phone	Planning to move outside the Providence/Chicago area within 1 year
6) Lived in the Providence/Chicago area ≥ 3 months	

### Study assessments

At baseline and follow-up, participants complete a staff-administered assessment and a computer-assisted self-interview system on an iPad. See Table 2 for assessment procedures at point of contact.

**Table 2:** Schedule of major assessment points

Point of Contact	Assessment Procedure(s)
Baseline/enrollment	Eligibility screening, informed consent, adherence assessment, comprehensive psychosocial assessment, viral load collection, Wisepill device training
Run-in visit (2-weeks post-baseline)	Group assignment, Wisepill acceptability survey
4-, 8-, 12-month follow-up visits	Adherence assessment, comprehensive psychosocial assessment, viral load collection

### Randomization

All participants complete a Wisepill “run-in” period two weeks following their baseline/enrollment visit and then a randomization visit at the end of this period. At this visit, participants are randomized to one of the two study groups. Block randomization, with blocks of four, are used for allocation at each site, with randomization assignment generated by computer. Block randomization ensures balanced representation in the two study groups at each site as recruitment progresses, with half of the sample randomized to the Positive STEPS intervention and half randomized to the SOC.

### Description of the intervention

The intervention is grounded in the social-cognitive and contextual realities (e.g., participants’ living circumstances, parental support, school situation) of HIV-infected adolescents and informed by social cognitive theory (SCT). SCT specifies a core set of mechanisms that influence health behavior with a primary emphasis on self-regulation (i.e., the accuracy and consistency of self-observation and self-monitoring) and self-reflection, including self-efficacy (i.e., the belief that one can exercise control over the health behavior of interest) [41, 42]. Text messages, which are sent daily to participants in the intervention, act as supporting external influences, are expected to enhance self-regulation, including self-efficacy to take medications as prescribed. The individual, face-to-face counseling sessions also address components of social cognitive theory such as motivation to take medications as prescribed and self-efficacy to do so through therapeutic strategies that employ cognitive-behavioral therapy and problem-solving therapy. Together, the receipt of text messages and the individualized counseling are expected to help overcome key barriers to adherence, including forgetting to take medication, as well as adherence self-efficacy, motivation and importance.

The Positive STEPS intervention uses an efficient stepped-care (“adaptive”) model [35] in which the least resource intensive intervention is delivered first, and only those individuals who do not improve then receive the high intensity, more resource intensive intervention. In the first step, participants randomized to the intervention receive low-intensity, daily, personalized, two-way texts messages for 12 months; these messages serve as social-cognitive cues or reminders to take their medications according to their medication schedule. A structured interview is used to personalize the messages to the participant’s needs, including the timing of dosage(s) and content of the message (e.g., to focus on reasons why taking HIV medications as prescribed is important to each participant). If participants demonstrate ≥90% adherence they will remain on Step 1. Participants who continue to have difficulty adhering to their HIV medications (< 90% adherence) anytime up to the end of month three (weeks 5–12) will progress to Step 2.

In Step 2, participants receive a manualized intervention consisting of five in-person counseling sessions, each lasting approximately 50 min. Each session incorporates adolescent-specific adherence counseling, digital video vignettes, and is delivered by a master’s-level counselor. In each counseling session, the patient and counselor define the adherence problem and challenges, generate alternative solutions, make decisions about the alternatives, determine the optimal solution, and develop an action plan (or update it) with skills training to implement that solution. The action plan includes setting goals, making a plan, discussing ART adherence skills, and monitoring adherence as a means of “self-regulation.” Each session begins with a discussion of participant’s adherence over the prior week (since their last visit), and reemphasizes the continued need for improved adherence in order to increase adherence motivations. When appropriate, the counselor engages in a discussion with participants about how drugs/alcohol may interfere with adherence as well as problem solving situations involving drug/alcohol use, establishing a plan to manage these challenges, and providing referrals to substance abuse treatment. During the sessions, the counselor and participant discuss how substances are used (frequency, context, setting, alone/with friends/with sexual partners/others, other’s use around them) and determine if substance use has been a barrier to optimal ART adherence for the participant.

Together, the counselor and participant identify an adherence plan that is both feasible and realistic. Based on the individualized plan, the counselor provides training in skills building and, when appropriate, practices newly learned behavioral skills with the participant (e.g., communicating effectively with peer pressure). All participants are provided referrals to adolescent and young adult mental health services, drug/alcohol treatment, and/or other adolescent HIV services in their area. See Table 3 for a summary of the topics addressed at each of the five counseling sessions.

**Table 3:** Summary of Positive STEPS counseling topics and video vignettes

Session	Topics Addressed	Video(s) Viewed
1: Introduction	- “Getting to know you” - Adherence psycho-education - Session roles & expectations	None
2: Logistics & HIV Care	- Getting to appointments - Getting medications - Talking with treatment team	“*Taking notes & asking questions at the doctor*”
3: Coping with HIV	- Side effects - Daily medication schedule - Managing mood	“*Trying to stay on a medication schedule*”
4: Privacy, Support, & HIV	- Managing social life - Dealing with disclosure - Storing medications	“*HIV-related stigma*” and “*Debate about disclosing HIV status*”
5: Adherence Slips & Wrap-up	- Taking care of “my own” HIV - Handling medication slips - Review of strategies that have and have not worked	“*Multiple strategies for adherence*”

### Description of the control condition

The control condition includes standard health services offered at each site (e.g., mental health services, case management) and a brief adherence educational session. The standard educational session consists of a review of medications and recommended dosing (i.e., to understand regimen), adherence expectations, toxicity expectations, and medication misperceptions. During the educational session, participants have the opportunity to ask questions and receive answers to reinforce their current ART regimen. The participant then views a 20-min animated tutorial that explains the importance of adherence to antiretroviral medication effectiveness. This video is specifically designed for to be viewed by individuals who have no scientific background and is appropriate for adolescents and young adults. The video, which is titled, “HIV Drug Resistance and the Importance of Adherence,” was created by BioCreations, a digital media company, in collaboration with the Johns Hopkins Point of Care Information Technology Center. The educational session is implemented during the group assignment visit, following randomization. All participants receive referrals to mental health, drug/alcohol treatment, and/or other adolescent HIV services as necessary.

### Primary outcome measures

#### Wisepill adherence

Wisepill is an innovative electronic medication monitoring device that is used to characterize patterns of ART adherence over the study period for both study groups. It is designed to be discrete and easy to transport. The Wisepill makes use of mobile phone and Internet technologies to provide real-time medication monitoring. Using Wisepill data, the percent of doses is calculated by dividing the number of times the device is opened by the number of times it should have been opened per the participant’s ART prescription. Potential Wisepill malfunctions are closely monitored and corrected by our trained team. Participants are asked to keep a log of doses that are taken without opening the Wisepill (i.e., “pocketed doses”). The log is then reviewed with study staff at each assessment visit and the log of Wisepill data is updated to reflect pocketed doses.

#### Self-report adherence

Items assessing self-reported adherence in the past 30 days and 4-months supplement the electronic monitoring data.

#### HIV viral load

One tube of blood will be drawn at each study assessment (baseline, 4-, 8-, and 12-months) to test for viral load.

### Secondary outcome measures

Quality of life and role impairment are assessed with a modified version (ACTGSF-21) of the SF-21, which is the quality of life measure used in AIDS Clinical Trials Group (ACTG) trials [43]. We also utilize the Quality of Life Inventory [44], which assesses life-satisfaction and differentiates role impairment, physical quality of life, and psychological quality of life. HIV symptoms and management are assessed with the Sign and Symptom Checklist HIV (SSC-HIVrev) [45]. Engagement in HIV care and medication changes for ART are also recorded at all assessment points.

### Mediators

Several mediators are hypothesized to explain the mechanisms through which the intervention is anticipated to improvement ART. Hypothesized mediators include: social support, which is assessed using items from the ACTG outcomes committee assessment and measures social support for adherence [46]. The HIV Medication Taking Self-Efficacy Scale is also used to measure ART adherence self-efficacy, or the confidence to take medications in various situations [47]. Motivation and outcome expectancies of ART adherence are assessed as three separate dimensions: attitudes, norms, and behavioral intentions to adhere to ART medication [48]. Self-regulation skills, which include self-monitoring, goal-setting, and enlistment of self-incentives/plans, are also assessed [49].

### Moderators

Several moderators are hypothesized to affect the strength of the intervention to yield improvements in ART adherence and will be explored. Hypothesized moderators include: depression as assessed via the 20-item Center for Epidemiologic Studies Depression Scale (CES-D) [50] and anxiety, which is assessed using the Beck Anxiety Inventory [51]. Problem alcohol and drug use are also assessed with the Alcohol, Smoking & Substance Involvement Screening Test (ASSIST) [52]. The ASSIST consists of nine items, covering ten substances (used in the past four months), including: tobacco, alcohol, cannabis, cocaine, stimulants, inhalants, sedatives, hallucinogens, opioids, and other drugs. The ASSIST assesses frequency of use and associated problems for each substance with good to excellent reliability and validity. In prior studies of adherence among adolescents, HIV-related stigma and discrimination has been strongly associated with non-adherence [53]; thus, we also measure HIV-related stigma with a ten-item validated scale created and tested specifically for adolescents (i.e., shortened to reduce participant burden) [54].

### Statistical analysis

The distribution of all variables will be assessed, as will the correlations between all variables and the primary/secondary outcomes. We will assess for patterns of missing data, which are expected to be low as the study utilizes CASI for sensitive data, which reduces participant nonresponse. If missing data are extensive, we will consider utilizing multiple imputation methods [55, 56]. The primary anticipated reason for missing data is attrition due to loss to follow-up. Based on our preliminary studies, we are accounting for 20% attrition from randomization to the 12-month assessment, and hence will enroll 192 participants (96 per study site) to achieve 160 completers (80 per site; 40 completers per arm, per site).

For all analyses (primary and secondary outcomes), we will use generalized linear models (GLM) with properly-chosen (based on the distribution of dependent variable) link functions to analyze longitudinal data for each major study aim. The GLMs will be estimated using generalized estimating equations with robust standard error estimates to account for repeated measures of the outcome. For each outcome, we will test the effects of the Positive STEPS intervention, compared to the comparison condition, at the 4-, 8- and 12-month follow-up time points. All statistical tests will determine significance with alpha = 0.05. To reduce the threat of bias, the intent-to-treat principle will be utilized, where cases will be analyzed according to the condition that they were randomized to. Participants in Step 2 who miss more than two sessions will be categorized as “non-completers” and analyzed secondarily in sensitivity analyses (dose-response, “as treated” analysis).

#### Primary outcome (aim 1)

The primary analysis will compare changes in adherence scores (continuous measure assessed via Wisepill) and viral load from baseline to the 4-, 8-, and 12-month visits between the randomized conditions. We will then replicate the main outcomes analysis with the self-reported adherence indicators and examine whether either indicator of adherence has a stronger association with changes in viral load.

#### Secondary outcomes (aim 2)

If the intervention arm shows improved adherence in greater magnitude than the comparison condition, we will explore the extent to which this relationship works through several possible mediators, including social support, self-efficacy, self-regulation, and motivation / outcome expectancies. For mediation analyses, we will employ MEDIATE procedures [52]. MEDIATE estimates the total, direct, and indirect effects of causal variable(s) (xlist) on the outcome variable (yvar) through a proposed mediator variable or set of mediator variables (mlist), controlling for (optional) one of more variables in (covlist). MEDIATE is similar to INDIRECT [57] but allows multiple X variables and also offers features for handling and coding a single multicategorical X variable. Inferences for indirect effects can be based on either percentile bootstrap confidence intervals or Monte Carlo confidence intervals. For effect modification analyses, we will add interaction terms one-by-one for the intervention condition and the potential moderators (e.g., psychosocial factors, such as depression). Significant or large interaction terms suggest that the effects of the intervention differ for different subgroups, as defined by the moderators.

### Sample size calculation

The primary power analysis is based on the acute ART adherence outcome (differences between the intervention and control conditions) from our Positive STEPS pilot RCT [20]. Given adherence to medications are actively monitored for participants in both arms using Wisepill, we expect the control group to have slightly improved adherence as well. We therefore powered the study to detect a 10% or greater difference in the rate of change in adherence between groups over the course of the study, expecting both groups to show improvements from baseline. Group sizes of 80 adolescent completers per arm (experimental intervention and the standard of care comparison), which assumes 20% attrition, will give greater than 90% power to detect at least a 10% or greater difference in adherence at 4-months in a pair-wise comparison.

## Discussion

We describe herein the design of the Positive STEPS study, a randomized controlled efficacy trial of a stepped-care, counseling- and technology-based intervention to improve ART adherence among HIV-infected adolescents with adherence difficulties. The design of this study has several strengths including, its focus on HIV-infected youth; the integration of technology with counseling to improve ART adherence; and the strength of the research design.

The Positive STEPS intervention is particularly innovative as it is designed for an increasingly young HIV-infected population with documented medication adherence challenges for which there is dearth of effective interventions. Recognizing the importance of a developmentally-tailored approach to improving health behaviors, the Positive STEPS intervention was informed, developed, and refined with input from the adolescent target population over a period of six years prior to testing the efficacy of the intervention in the current trial [19, 20]. This iterative, community-engaged approach ensured that the intervention content is inclusive of adolescents’ contextual realities and that these realities are addressed in a manner that promotes ART adherence skills building and problem solving as a means of improving ART adherence, health and quality of life through intervention components that are acceptable to youth.

The study is also novel in its use of a stepped-care intervention design and the employment of technological approaches to maximize ART adherence. Specifically, the adaptive, stepped-care approach used here, allows us to test an efficient and real-world approach to HIV medication adherence in which the least resource intensive intervention is delivered first, and only those who do not improve receive the high-intensity, more resource intensive intervention. Further, the integration of two-way text messaging allows youth to receive intervention components outside the clinical setting and facilitates adherence discussions between adolescents and their counselors through technology-based forms of communication that are highly acceptable to youth [19]. The employment of digital video vignettes further enhances the intervention as they bring to life HIV-specific scenarios that youth can relate to and that can be accessed and viewed by participants via their mobile phones or computers on their own time. Finally, the assessment of adherence using electronic, self-report, and biological adherence measures provides multiple reference points for analyzing intervention effects.

Given the absence of effective, developmentally-tailored and rigorously tested ART adherence interventions for youth in the published literature, to our knowledge, this study is the first intervention of its kind to be tested in a rigorous trial of sufficient size to detect improvements in ART adherence in a population of HIV-infected youth. This study was designed to meet the CDC standard for evidence-based interventions and to extend the base of evidence for intervention for a group with the highest rates of HIV. If shown to be efficacious, this intervention will be ready for dissemination via a translational effectiveness trial with the ultimate goal of making the intervention available to counselors in diverse settings in order to improve ART adherence among HIV-infected youth and reduce the spread of HIV.

## Abbreviations

ACASI: Audio computer-assisted self-interview
ACTG: AIDS Clinical Trials Group
ART: Antiretroviral therapy
CDC: Centers for disease control and prevention
HIV: Human immunodeficiency virus
RCT: Randomized controlled trial
SCT: Social cognitive theory
SOC: Standard of care
